# Automatic Measurement of Morphological Traits of Typical Leaf Samples

**DOI:** 10.3390/s21062247

**Published:** 2021-03-23

**Authors:** Xia Huang, Shunyi Zheng, Li Gui

**Affiliations:** 1 School of Remote Sensing and Information Engineering, Wuhan University, Wuhan 430079, China; yuxiuhx@whu.edu.cn (X.H.); whuguili@whu.edu.cn (L.G.); 2 Collaborative Innovation Center of Geospatial Technology, Wuhan 430079, China

**Keywords:** plant measurement, morphological traits, 3D plant model, segmentation

## Abstract

It is still a challenging task to automatically measure plants. A novel method for automatic plant measurement based on a hand-held three-dimensional (3D) laser scanner is proposed. The objective of this method is to automatically select typical leaf samples and estimate their morphological traits from different occluded live plants. The method mainly includes data acquisition and processing. Data acquisition is to obtain the high-precision 3D mesh model of the plant that is reconstructed in real-time during data scanning by a hand-held 3D laser scanner (ZGScan 717, made in Zhongguan Automation Technology, Wuhan, China). Data processing mainly includes typical leaf sample extraction and morphological trait estimation based on a multi-level region growing segmentation method using two leaf shape models. Four scale-related traits and six corresponding scale-invariant traits can be automatically estimated. Experiments on four groups of different canopy-occluded plants are conducted. Experiment results show that for plants with different canopy occlusions, 94.02% of typical leaf samples can be scanned well and 87.61% of typical leaf samples can be automatically extracted. The automatically estimated morphological traits are correlated with the manually measured values EF (the modeling efficiency) above 0.8919 for scale-related traits and EF above 0.7434 for scale-invariant traits). It takes an average of 196.37 seconds (186.08 seconds for data scanning, 5.95 seconds for 3D plant model output, and 4.36 seconds for data processing) for a plant measurement. The robustness and low time cost of the proposed method for different canopy-occluded plants show potential applications for real-time plant measurement and high-throughput plant phenotype.

## 1. Introduction

Leaf morphological traits are indices describing plant shape and architecture. They can be used in many fields of agriculture research, such as plant growth and health condition monitoring [1], plant modeling [2], agricultural simulation [3], farm management decision-making design [4], high-throughput plant phenotype analysis [5], and genome-wide association study [6]. The sample leaves are mainly selected based on experience in traditional leaf trait measurement, which is time-consuming and cumbersome. In some agricultural research, such as high-throughput phenotype, a large number of samples are needed [7]. The automatic sample selection has become necessary. Nevertheless, various sensors, such as the color digital cameras, range cameras, hyperspectral cameras, multispectral cameras, thermal imagers, infrared radiometers, fluorescence sensors, light detection and ranging (LIDAR) sensors, global positioning system (GPS) receivers, and laser sensors have been used for plant measurement [8]. Those sensors can generate a large amount of data. Traditional leaf trait measurement that mainly relies on manual measurement methods based on contact tools is not suitable for such a large capacity of data processing. Automatic morphological trait estimation should be carried out and automatic data batch processing is urgently needed. Moreover, the leaf morphological structures of different species are different, which makes the calculation of leaf morphological traits vary. Therefore, it is significant to study automatic measurement of morphological traits of typical leaf samples.

Image technology has been widely used in plant measurement. An et al. [9] developed an imaging system and proposed a series of image processing algorithms to construct two-dimensional (2D) mosaicked orthophotos. Morphological traits such as leaf length and rosette area were measured by these 2D top-view images. Fanourakis et al. [10] used a phenotypic platform light curtain array (LC) to evaluate leaf area and maximum height from side-view profile images. Pereyrairujo et al. [11] introduced a low-cost platform for high-throughput measurement of plant growth. This imaging approach estimated morphological traits based on 2D images. The top-view and side-view images were required to ensure measurement accuracy when measuring leaf morphological traits such as area, perimeter, length, and width. If the image was shot at random angles, the measurement results would deviate greatly from the true values.

Since the aforementioned works based on 2D imaging technology not only require images with special shooting angles but also lose part of the 3D information, 3D technology that can accurately measure the 3D structure of plants is rising rapidly. Biskup et al. [12] used an area-based binocular stereo imaging system to build a 3D plant model and measure leaf inclination angles. Gibbs et al. [13] adopted the structure-from-motion (SFM) technique to build a 3D plant model. This method required a large number of images and cost a lot of time to reconstruct the 3D model. Kjaer et al. [14] employed a 3D NIR-laser scanner to track daily changes in the growth stages of different plants in challenging environments. The scanner was fixed and the 3D model of the plant was incomplete. Xia et al. [15] used a Kinect RGB-D camera to measure individual plant leaves. Mean shift clustering was employed to segment individual leaves from the background. However, no morphological traits were estimated. Drapikowski [16] employed a structured-light DAVID to estimate the morphological traits of Xerophytic plants. The morphological traits such as length, width, and area were automatically estimated based on rough plant models. Martínezguanter et al. [17] used a LiDAR sensor to detect plant spacing. A large number of 3D models of plants were reconstructed. The point cloud data obtained by Kinect, David, and LiDAR were usually rough, so it was difficult to reconstruct high-precision 3D plant models. These methods usually obtained 3D points first, and then the 3D plant model was reconstructed to measure morphological traits. However, these models were usually very rough and lacked detailed geometry information, which would affect the measurement accuracy. Thus, it is significant to obtain high-precision 3D models of plants.

Furthermore, most contemporary studies of leaf trait measurement are mostly limited to the plants without canopy occlusion at the early growth stage, and all leaves are counted [18,19]. Plants with heavy occlusion are rarely explored in individual leaf trait measurement research. More leaves mean heavier occlusion problems, which makes adequate data acquisition difficult and automated data analysis challenging. However, canopy occlusion is common in reality. Therefore, it is practically significant to measure individual leaf morphological traits of plants with different canopy occlusions. Additionally, most contemporary research of leaf measurement involves all leaves on the plants. In research such as leaf classification and plant species identification, typical leaf samples do not include the curved new-born leaves, damaged leaves, or rotten leaves [20,21]. Therefore, it is worthwhile to study the automatic selection of typical leaf samples. Nevertheless, the morphological structures of leaves are different. Specifically, leaves of plants, such as corn [22,23,24], rice [25], and wheat [26], are long and banding. Leaves of plants, such as Epipremnum aureum [27], Anthurium Andraeanum [28], and leafy vegetables [29], are broad and elliptical. The calculation algorithms of their morphological traits, especially length and width, are different. Additionally, leaf morphological traits in the aforementioned research are usually leaf area, length, and width. Those traits are scale-related. More scale-invariant traits should be explored.

Thus, research of new methods that employs 3D technology for automatic plant measurement is of great interest. The automatic data acquisition and processing methods that can perform high-throughput phenotyping functions with adequate precision requirements are useful in modern agriculture. The objective of this study is to solve three problems, namely, the acquisition of the high-precision 3D plant model, automatic selection of typical leaf samples, and automatic estimation of leaf morphological traits. In this paper, we propose a novel method to automatically select typical leaf samples and estimate their morphological traits from different canopy-occluded plants based on a hand-held 3D laser scanner. The method mainly includes two parts: (1) data acquisition with a hand-held 3D laser scanner to obtain the 3D mesh model of the plant, and (2) the multi-level region growing segmentation using two leaf shape models to automatically select typical leaf samples and estimate their morphological traits. Particularly, the 3D plant model can be reconstructed in real-time during data scanning. When segmenting individual leaves of different plants at different scales, the two main segmentation parameters, smooth and curvature, are adaptive. Two shape models, one using the scale-related traits and the other employing the scale-invariant traits, are dynamically established based on the segmentation results at different scales using the principal component analysis method. Four scale-related morphological traits and six corresponding scale-invariant traits are automatically estimated. Epipremnum aureum, one of the most common plants with broad and elliptical leaf structure, is adopted as the experiment subject. The proposed method will be tested on four groups of Epipremnum aureum with different canopy occlusions. The accuracies of the data scanning, segmentation, and trait estimation will be analyzed.

## 2. Materials and Methods

### 2.1. Data Acquisition and Processing Environment

The data acquisition was performed using a ZGScan 717 (parameters listed in Table 1) hand-held 3D laser scanner in Wuhan, China. The time was November 2020. The data acquisition was conducted indoors. There were four groups of Epipremnum aureum with different canopy occlusions. Each group contained three plants. Plants in each group had similar canopy occlusion. The occlusion increased from the first group to the fourth group. Specifically, the plants of the first, second, third, and fourth groups had no, a little, medium, and heavy canopy occlusion. The plants in each group were scanned individually.

**Figure 1:**
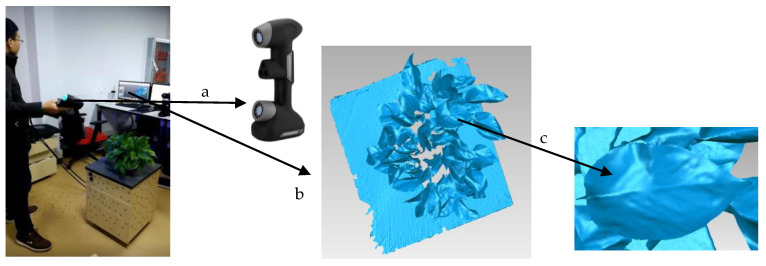
Data acquisition process. (**a**) ZGScan 717; (**b**) The generated 3D mesh model of the plant; (**c**) The 3D mesh model of a leaf.

The plant was on the table and scanned by ZGScan 717 (Figure 1a). With two CCDs and one laser, ZGScan 717 recorded data on a contour section of an object surface by projecting seven pairs of cross scanning laser beams and one deep hole scanning laser beam. To reconstruct the 3D model of the plant, the position and orientation of the detection handle were detected by the embedded motion-tracking technology. It quickly scanned the object (480,000 times/second) and generated high-precision point clouds. The maximum accuracy could reach 0.03 mm. ZGScan 717 was held close to the plant during data scanning with no closer than 80 mm and no distant than 2000 mm to ensure a high-resolution scanning result. The scanning path was random. The 3D model was reconstructed in real-time when data scanning. Particularly, the reconstructed 3D model was viewed on a laptop in real-time when data scanning, which helped to check the integrity of the data acquisition. Specifically, complex parts of the plant should be scanned more carefully. Particularly, the software integrated with ZGScan 717 could output both 3D point clouds and 3D mesh. Here, the 3D mesh was chosen as the scanning output (Figure 1b). Figure 1 shows the data acquisition process. As shown in Figure 1b,c, the output 3D mesh model of the plant is high-precision with detailed geometric structure.

The data processing algorithms were implemented on a 2.50 GHZ desktop with 8.0 Gb RAM. The code was compiled using C++ with Point Cloud Library (PCL) v1.8.0, Computational Geometry Algorithms Library (CGAL), and Visualization Toolkit (VTK) v7.0. All algorithms were integrated. The entire process of data processing was conducted by algorithms without manual works.

### 2.2. Typical Leaf Sample Extraction and Morphological Trait Estimation

Individual leaves should be segmented to measure leaf morphological traits. A multi-level region growing segmentation method based on the leaf shape model is proposed. The region growing method divides the point clouds into different clusters mainly based on smooth and curvature characters [30]. Points with similar smooth and curvature values are segmented into a group. Different smooth and curvature thresholds can lead to different results. Segmentation at a large scale based on the region growing method will lead to a rough segmentation result, and if the segmentation scale is too small, it may be over-segmented. When the input data varies, the most appropriate scale should be different. However, the segmentation scale and its parameters are usually set by experience in most contemporary research. The goal of our proposed segmentation method is to find a suitable adaptive segmentation scale for different input data. Considering that leaves in the same species have similar morphological structures, the shape model is employed in segmentation. The proposed segmentation method mainly includes six steps, as shown in Figure 4.

**Figure 2:**
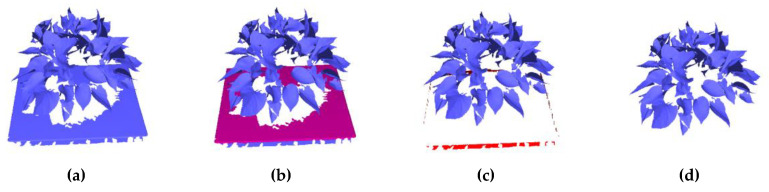
The removal of non-plant. (**a**) An example of the generated 3D mesh model of the plant; (**b**) the detection of the table; (**c**) the filtering of non-plant; (**d**) the plant after the non-plant removal.

Step 1: Non-plant removal. The main purpose of non-plant removal is to remove the plant from the background. Here, the RANSAC [31] plane detection method is adopted to remove the table, as shown in Figure 2b. Then we filter out the triangles with a distance range of *ε* (*ε* = 100 mm) from the table, as shown in Figure 2c. Those data are usually the table, flower pot, and the soil. At last, plant data are extracted from the background (Figure 2d). Particularly, some data of the flower pot and the soil may be missing in some experiments, as they are not our target and are not scanned properly during the scanning process, as shown in Figure 2.

Step 2: Region growing segmentation. Smooth and curvature are two decisive parameters for region growing segmentation. The most important step is to find the appropriate values of smooth and curvature. The average of the smooth and curvature, ς and ρ, are calculated first. The number of points *N_p_* for fitting a local surface and calculating curvature is usually 30. The values of smoothness and curvature thresholds *ε*_a_ and *ε*_b_ in the initial segmentation scale are *ε*_a_ = 1.5 ς and *ε*_b_ = 1.5 ρ.

Step 3: Estimation of morphological traits. Assume all the segmentation results are leaves. All morphological traits of each segmentation result are calculated. Typical shape-based leaf morphological traits include scale-related and scale-invariant traits. Generally, leaf area, perimeter, length, and width are scale-related morphological traits, and their ratios are scale-invariant morphological traits, as listed in Table 2.

**Figure 3:**
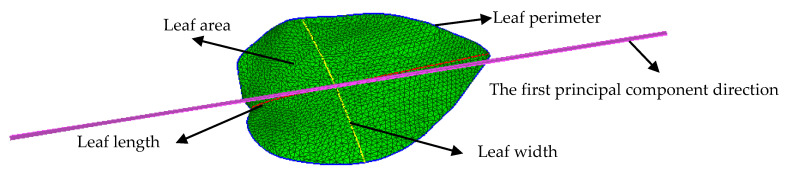
Visualization of four scale-related morphological traits (leaf area, perimeter, length, and width) on the 3D-triangle mesh of a leaf.

**Figure 4:**
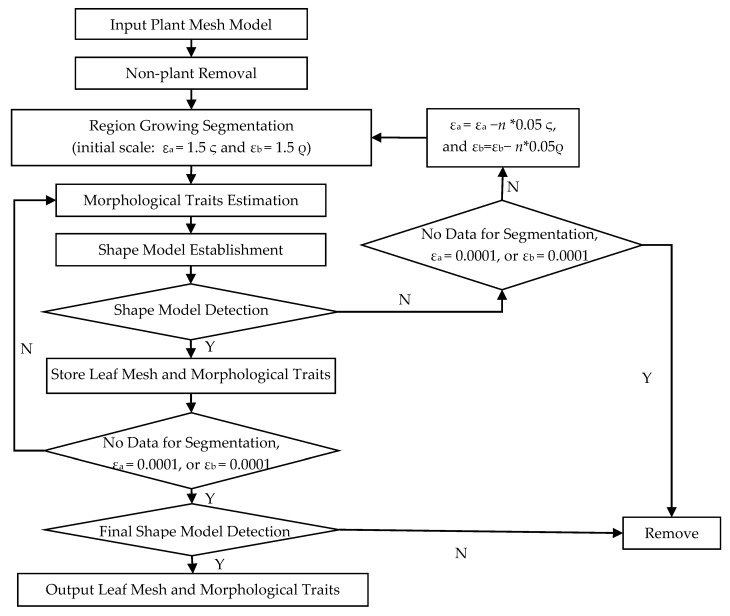
The multi-level region growing segmentation method based on the shape model.

Leaf area (*s*) is an important scale-related trait. As shown in Figure 3, the leaf area can be represented by a triangular mesh (green mesh). Leaf perimeter (*c*) is the boundary of the mesh (blue line in Figure 3). The length of the boundary is the leaf perimeter. Leaf length (*l*) is usually measured along the midline of the leaf. The shape of the leaf is broad and elliptical; thus, the longest shortest geodesic path along the first principal component direction (purple line in Figure 3) is the target leaf length (red line in Figure 4). Leaf width (*w*) (yellow line in Figure 3) is usually defined as the widest length of the leaf perpendicular to the leaf length.

Step 4: Establishment of the shape model. Two shape models are established to describe the leaf parametrically. One is used for size description and the other is employed for architecture description. The formulas for these two shape models are as follows:

(1) $$F(X)=a_{1}\frac{X_{01}-\bar{X_{01}}}{\bar{X_{01}}}+a_{2}\frac{X_{02}-\bar{X_{02}}}{\bar{X_{02}}}+a_{3}\frac{X_{03}-\bar{X_{03}}}{\bar{X_{03}}}+a_{4}\frac{X_{04}-\bar{X_{04}}}{\bar{X_{04}}}$$

(2) $$G\left(X\right)\;=\;b_{1}X_{11}+\;b_{2}X_{12}+\;b_{3}X_{13}+\;b_{4}X_{14}+\;b_{5}X_{15}+\;b_{6}X_{16}$$

where *a*_1_–*a*_4_ and *b*_1_–*b*_6_ are variance contribution rates of the principal component analysis of the scale-related traits and scale-invariant traits, respectively. *X*_01_～*X*_04_ and *X*_11_～*X*_16_ are traits in Table 2.

Step 5: Detection of the shape model. A series of clusters are obtained after the initial segmentation. Some leaves can be successfully segmented, while some attached and overlapped leaves may be segmented incorrectly. Therefore, correct results should be stored, and incorrect clusters should be further segmented. If a segmented result satisfies:

(3) $$H\left(X\right)=\left\{\begin{array}{l} 0.25*\bar{F\left(X\right)}<F\left(X\right)\;<1.25*\bar{F\left(X\right)}\\ 0.25*\bar{G\left(X\right)}<G\left(X\right)\;<1.25*\bar{G\left(X\right)} \end{array}\right.$$

The segmented result is a typical leaf sample, and its mesh and morphological traits are stored. If not, repeat step 2 with a smaller segmentation scale (smaller values of *ε*_a_ and *ε*_b_). *ε*_a_ and *ε*_b_ are reduced by 5% in each cycle. Repeat this process until all segmentation results meet the shape model detection or one of *ε*_a_ and *ε*_b_ is reduced to 0.0001.

Step 6: Final shape model detection and the outputs. When the segmentation is done, detect all the stored segmentation results with the final shape model that is based on all the stored segmentation results. If a segmentation result satisfies the final shape model, the 3D leaf model and their morphological traits are output.

**Figure 5:**
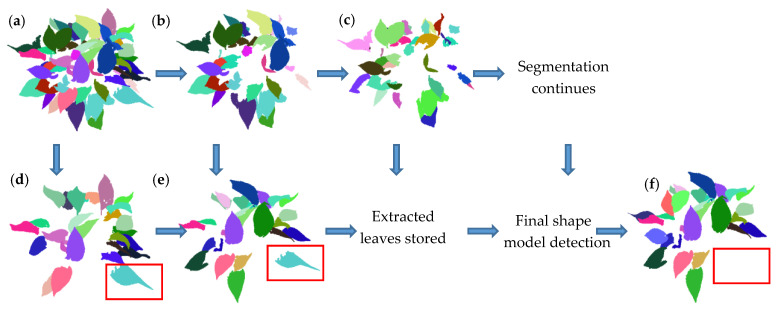
Schematic diagram of the multi-level region growing segmentation. (**a**) Initial segmentation results (*ε*_a_ = 1.5ς and *ε*_b_ = 1.5ρ); (**b**) the data for the second segmentation; (**c**) the data for the third segmentation; (**d**) the automatically selected leaves after the initial segmentation based on the initial shape models; (**e**) the automatically selected leaves after the second segmentation (*ε*_a_ = 1.45ς and *ε*_b_ = 1.45ρ) based on the second shape models; (**f**) the automatically selected typical leaf samples after the final segmentation based on the final shape models. The red boxes mark a cluster considered as a typical leaf sample during the multi-level segmentation process but removed at last.

The multi-scale segmentation method proposed in this paper is an iterative process. Rough segmentation results C__level1_ are usually obtained through the initial segmentation (*ε*_a_ = 1.5ς and *ε*_b_ = 1.5ρ), as shown in Figure 5a. The first shape model H(X) __level1_ is built based on all clusters in C__level1_. Then all clusters in C__level1_ are detected by H(X) __level1_. Clusters in C__level1_ that satisfy the H(X) __level1_, defined as M__H(X)_level1_, are stored, as shown in Figure 5d. Clusters that do not satisfy the H(X) __level1_, defined as M__R_level1_, go through the second segmentation at a smaller scale (*ε*_a_ = 1.45ς and *ε*_b_ = 1.45ρ), as shown in Figure 5b. Then a new shape model H(X) __level2_ is built based on M__H(X)_level1_ and the second segmentation results C__level2_. Clusters in M__H(X)_level1_ and C__level2_ that satisfy the H(X) __level2_, defined as M__H(X)_level2_, update M__H(X)_level1_, as shown in Figure 5e. Clusters in C__level2_ that do not satisfy the H(X) __level2_, define as M__R_level2_, go through further segmentation, as shown in Figure 5c. The segmentation continues until all segmentation results meet the shape model detection or one of *ε*_a_ and *ε*_b_ is reduced to 0.0001. M__H(X)_leveln_ is defined as the clusters that satisfy the last shape model H(X) __leveln_ after the final segmentation. When the segmentation is done, a final shape model H(X) is built based on clusters in M__H(X)_leveln_. All clusters in M__H(X)_leveln_ are detected by H(X). Then clusters (M__H(X)_) that satisfy the H(X) are the typical leaf samples, as shown in Figure 5f. As more and more typical leaves are segmented correctly, the established shape model becomes more and more accurate, which in turn improves the automatic segmentation accuracy of typical leaves. A segmentation result may be considered as the typical leaf sample after the initial segmentation based on the initial shape model, while it will be removed in further shape model detection, as shown in the red box in Figure 5.

### 2.3. Accuracy Analysis

The accuracies of the data scanning, segmentation, and trait estimation are tested. The comparison between automatically and manually selected typical leaf samples is conducted to illustrate the accuracy of the segmentation. *R__scan_* is the data scanning accuracy. *R__seg_*_1_ is the segmentation accuracy between automatically segmented typical leaf samples and well-scanned typical leaf samples. *R__seg_*_2_ is the segmentation accuracy between automatically segmented typical leaf samples and manually selected typical leaf samples. *R__scan_*, *R__seg_*_1_, and *R__seg_*_2_ can be calculated as follows:

(4) $$R_{\_\mathrm{scan}}=\frac{N_{2}}{N_{1}}\times 100\%,R_{\_\mathrm{seg}1}=\frac{N_{3}}{N_{2}}\times 100\%,\;\mathrm{and}\;R_{\_\mathrm{seg}2}=\frac{N_{3}}{N_{1}}\times 100\%$$

where *N*_1_, *N*_2_, and *N*_3_ are the numbers of manually selected, well scanned, and automatically selected typical leaf samples. 

Correlation analyses between the automatically estimated and manually measured traits are performed to verify the accuracy of trait estimation. A dimensionless statistic that directly relates model predictions to observed data is the modeling efficiency (*EF*) [32]. *EF* is a statistic based on the coefficient of determination. The calculated *EF* is an overall indication of goodness of fit. If a model has a negative value of *EF*, the model cannot be recommended. If *EF* has preferable values close to one, it indicates a “near-perfect” model. *EF* is defined as:

(5) $$EF=1-\sum_{i=1}^{n}(y_{i}-x_{i}{)}^{2}/\sum_{i=1}^{n}(y_{i}-\bar{y}{)}^{2}$$

where *x_i_* is the independent variable, *y_i_* is the dependent variable, and *n* is the number of samples. In this paper, *x_i_* is the automatically estimated morphological trait and *y_i_* is the manually measured morphological trait. 

Additionally, the root-mean-square error (*RMSE*) and the mean absolute percentage error (*MAPE*) of the automatic measurement traits compared to the manually measured traits are adopted to illustrate the accuracy of trait estimation. The formulas for *RMSE* and *MAPE* are as follows:

(6) $$\mathrm{RMSE}=\sqrt{\frac{1}{n}\sum_{i=1}^{n}{\left(x_{mi}-x_{ai}\right)}^{2}}$$

(7) $$\mathrm{MAPE}=\frac{1}{n}\sum_{i=1}^{n}\frac{\left|x_{mi}-x_{ai}\right|}{x_{mi}}\times 100\%$$

where *x_mi_* is the manual measurement traits and *x_ai_* is the automatically estimated traits. 

## 3. Results 

### 3.1. Results of Data Scanning and Segmentation

The data scanning results and automatic segmentation results of typical leaf samples are shown in Table 3. The number of leaves *N*_0_ in the first and second group is 3–8 and 10–11. The number of typical leaf samples *N*_1_ in the first and second group is 3–6 and 8–11. The difference between *N*_0_ and *N*_1_ indicates that there are some new-born leaves or damaged leaves that are not suitable for morphological trait estimation. The scanning accuracy (R__scan_) and segmentation accuracies (R__seg1_ and R__seg2_) reach 100%. This indicates the data scanning accuracy with the hand-held scanner ZGScan 717 is competitive and the automatic segmentation method proposed in this paper is effective for plants with no or a little occlusion. For plants in the third group, *N*_0_ ranges from 26 to 34, and *N*_1_ varies from 24 to 31. This indicates there are new-born or damaged leaves. The values of R__scan_ (96.77–95.83%), R__seg1_ (95.83–93.33%), and R__seg2_ (92.00–90.32%) exhibit that the proposed segmentation method is practical for the plants with medium occlusion, although some data are missing. In the fourth group, *N*_0_ varies from 45 to 53, and *N*_1_ ranges from 36 to 37, which indicates there are a large number of new-born or damaged leaves. R__scan_ ranges from 94.59% to 86.11%. R__seg1_ varies from 91.43% to 87.10%. R__seg2_ is reduced from 86.43% to 75.00%. The values of R__scan_, R__seg1_, and R__seg2_ indicate that occlusion affects data scanning and segmentation accuracy a lot for the occluded plants. 

In general, for plants from the first group to the fourth group, the average data scanning accuracy R_1_ is 100–89.96% and segmentation accuracy R_2_ and R_3_ are 100–88.8% and 100–79.95%. R_1_, R_2_, and R_3_ decrease with the increase of canopy occlusion. The difference between R_2_ and R_3_ shows that the main factor that affects the segmentation results is the incomplete scanning data, which indicates the occlusion is the main factor that affects the segmentation accuracy. Generally, for all plants with different canopy occlusions, R__scan_, R__seg1_, and R__seg2_ are 94.02%, 93.18%, and 87.61%, respectively. 

**Figure 6:**
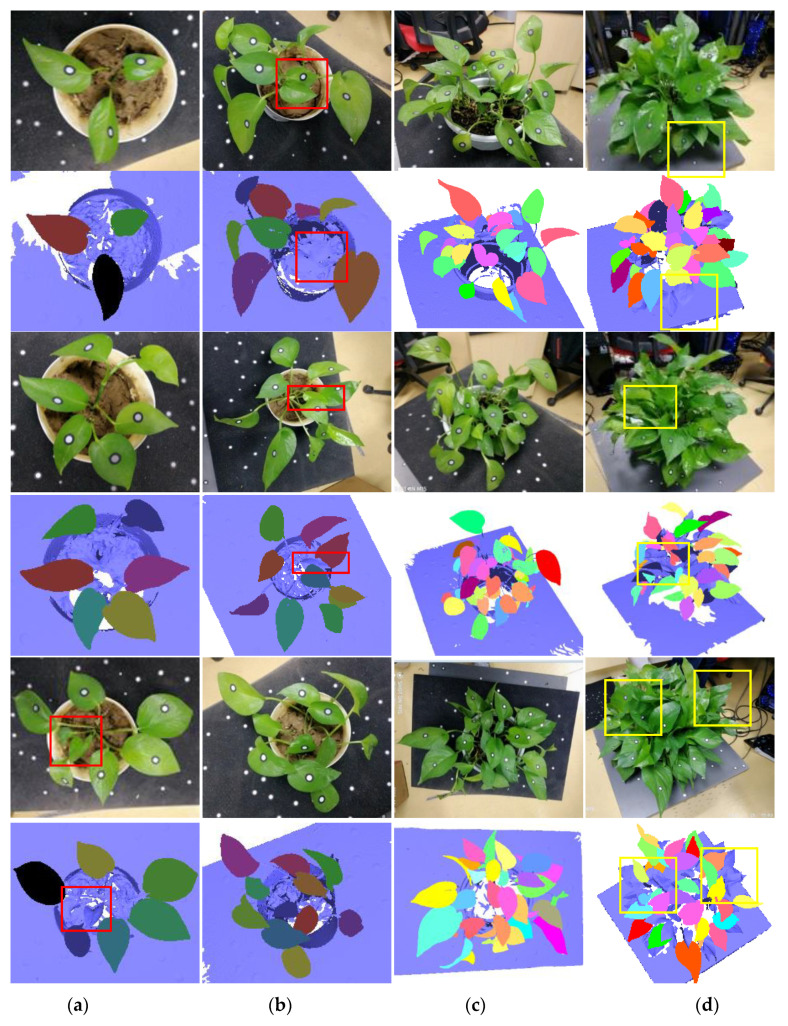
The automatic segmentation results of typical leaf samples of different canopy-occluded plants. (**a**) No canopy occlusion (group 1); (**b**) a little canopy occlusion (group 2); (**c**) medium canopy occlusion (group 3); (**d**) heavy canopy occlusion (group 4). The red boxes mark the new-born leaves, and the yellow boxes mark the leaves with incomplete scanning data.

Figure 6 shows the segmentation results of typical leaf samples of different canopy-occluded plants. Figure 6a–c show that the proposed method can successfully segment typical leaf samples from plants with no occlusion, a little occlusion, and medium occlusion. Small new-born leaves that are not suitable for morphological trait measurement, such as the leaves in the red boxes in Figure 6, are automatically removed. Figure 6d illustrates that most of the typical leaf samples can be successfully extracted from heavily occluded plants. Leaves with incomplete scanning data (the leaves in the yellow boxes in Figure 6) are automatically removed. The number of typical leaves that are not successfully extracted increases as the number of lost scanning data increases.

Table 3 and Figure 6 both show that the scanning and segmentation accuracy decreases with the increase of canopy occlusion. The increase of leaves and the aggravation of occlusion make the structure of the plant more complex, which increases the difficulty of data scanning and individual leaf segmentation. Specifically, on the one hand, the complex structure of the occluded plants directly affects the segmentation results; on the other hand, occlusion will affect the integrity of the scanning data and indirectly affect the segmentation accuracy.

### 3.2. Results of Morphological Trait Estimation

#### 3.2.1. Results of Scale-related Morphological Traits

Scale-related traits contain leaf area, perimeter, length, and width in this paper. The comparison between the automatically estimated and the manually measured scale-related traits are performed.

**Figure 7:**
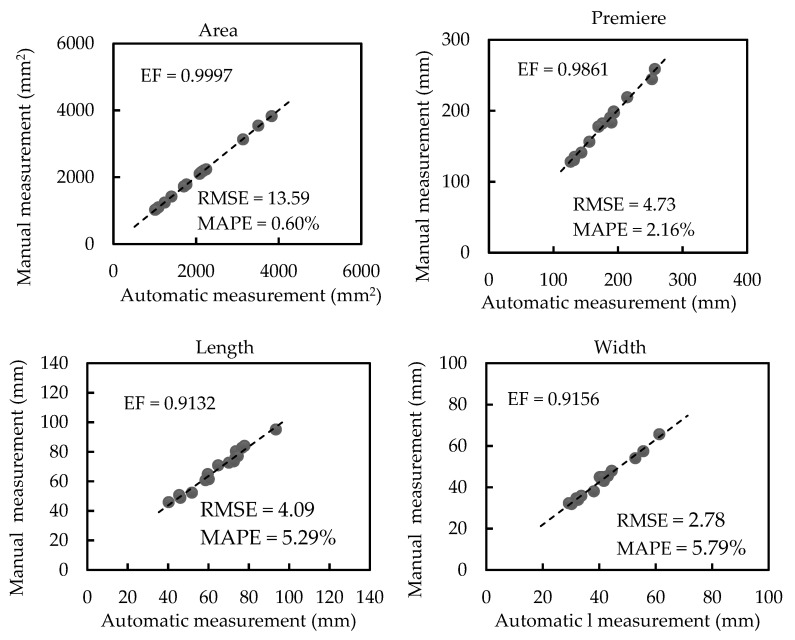
Regression analyses between automatic and manual measurements of scale-related traits of plants with no occlusion (plants in group 1).

Figure 7 illustrates the regression analysis between automatic and manual measurements of scale-related traits of plants with no occlusion (plants in group 1). The average values of leaf area, perimeter, length, and width are 2017.50 mm^2^, 180.65 mm, 65.03 mm, and 41.13 mm, respectively. Regression analyses show strong correlations (EF = 0.9997, EF = 0.9861, EF = 0.9132, and EF = 0.9156 for the area, perimeter, length, and width, respectively). Nevertheless, the RMSE values of the area, perimeter, length, and width are 13.59 mm^2^, 4.73 mm, 4.09 mm, and 2.78 mm, respectively. The MAPE values of area, perimeter, length, and width are 0.60%, 2.16%, 5.29%, and 5.79%, respectively. Regression analyses, RMSE, and MAPE results show that the deviation between automatic estimation and manual measurement is very small. 

**Figure 8:**
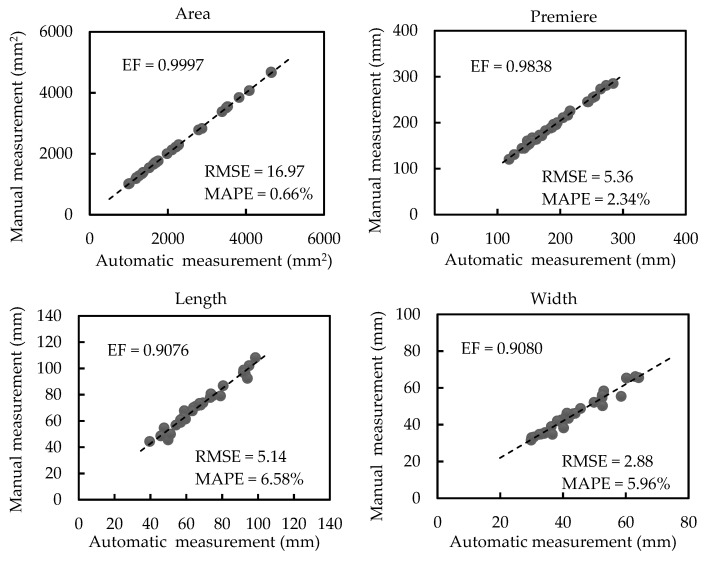
Regression analyses between automatic and manual measurements of scale-related traits of plants with a little occlusion (plants in group 2).

Figure 8 shows regression analyses between automatic and manual measurements of scale-related traits of plants with a little occlusion (plants in group 2). As shown in Figure 8, the average leaf area, perimeter, length, and width are 2260.46 mm^2^, 189.2 mm, 68.3 mm, and 43.34 mm, respectively. The automatically calculated values are highly correlated with the manually measured values based on the proposed method (EF = 0.9997, EF = 0.9838, EF = 0.9076, and EF = 0.9080 for the area, perimeter, length, and width, respectively). Additionally, the RMSE values of the area, perimeter, length, and width are 16.97 mm^2^, 5.36 mm, 5.14 mm, and 2.88 mm, respectively. The MAPE values of the area, perimeter, length, and width are 0.66%, 2.34%, 6.58%, and 5.96%, respectively. The regression analyses and the results of RMSE and MAPE exhibit little deviation of automatic measurement values from manual measurement values for plants with a little occlusion.

**Figure 9:**
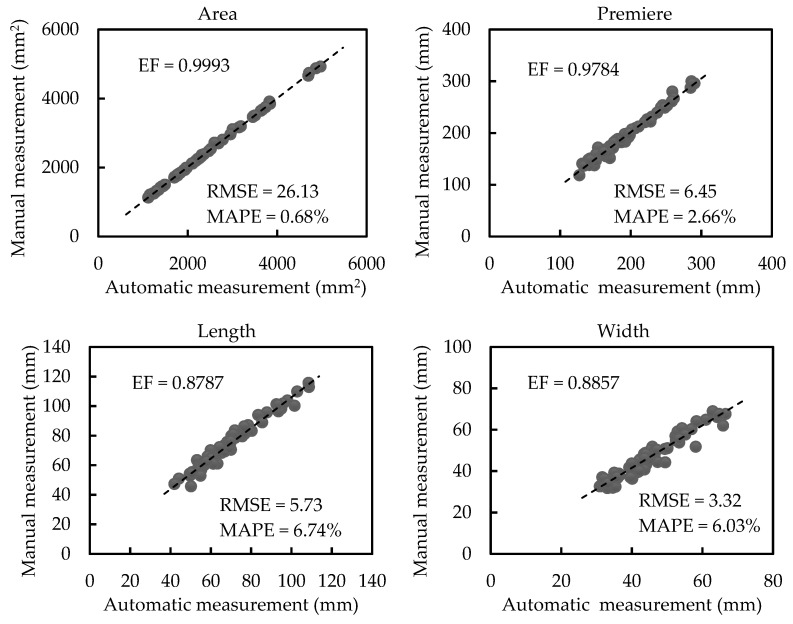
Regression analyses between automatic and manual measurements of scale-related traits of plants with medium occlusion (plants in group 3).

Figure 9 displays regression analyses between automatic and manual measurements of scale-related traits of plants with medium occlusion (plants in group 3). The leaf area, perimeter, length, and width have average values of 2270.66 mm^2^, 190.35 mm, 69.33 mm, and 44.71 mm, respectively. EF for the area, perimeter, length, and width, are 0.9993, 0.9784, 0.8787, and 0.8857, respectively. This indicates the automatically calculated values are highly correlated with the manually measured values. Furthermore, the RMSE values of the area, perimeter, length, and width are 26.13 mm^2^, 6.45 mm, 5.73 mm, and 3.32 mm, respectively, and the MAPE values of the area, perimeter, length, and width are 0.68%, 2.66%, 6.74%, and 6.03%, respectively. The regression analyses and the results of RMSE and MAPE exhibit a small deviation of automatic measurement values from manual measurement values for plants with medium occlusion. 

**Figure 10:**
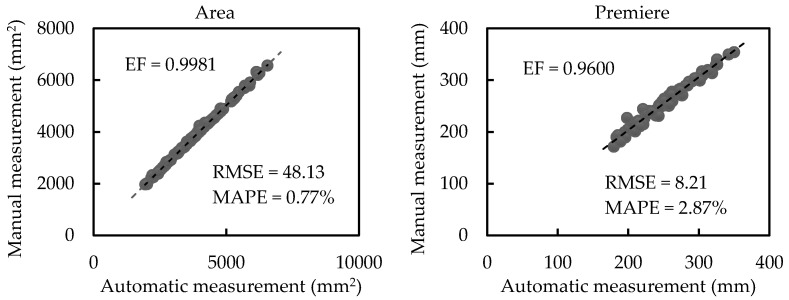

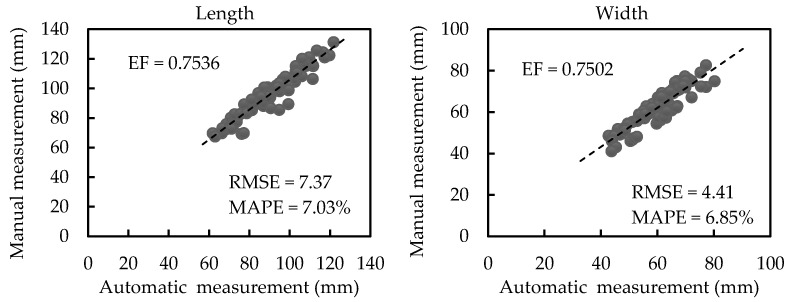
Regression analyses between automatic and manual measurements of scale-related traits of plants with heavy occlusion (plants in group 4).

Figure 10 exhibits regression analyses between automatic and manual measurements of scale-related traits of plants with heavy occlusion (plants in group 4). Meanwhile, values of RMSE and MAPE are exhibited. The average leaf area, perimeter, length, and width have values of 3845.10 mm^2^, 252.48 mm, 89.75 mm, and 59.06 mm, respectively. EF for area, perimeter, length, and width, are 0.9981, 0.9600, 0.7536, and 0.7502, respectively. This illustrates the automatic measurement values have strong explicit correlations with the manual measurement values. Moreover, the RMSE values of the area, perimeter, length, and width are 48.13 mm^2^, 8.21 mm, 7.37 mm, and 4.41 mm, respectively, and the MAPE values of the area, perimeter, length, and width are 0.77%, 2.87%, 7.03%, and 6.85%, respectively. The regression analyses and the results of RMSE and MAPE indicate a small deviation of automatic measurement values from manual measurement values for plants with heavy occlusion.

**Figure 11:**
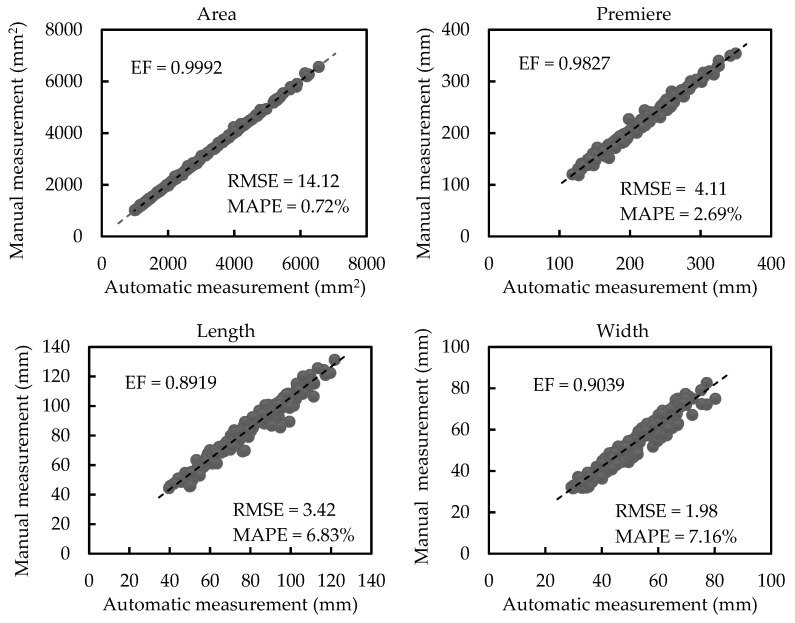
Regression analyses between automatic and manual measurements of scale-related traits of plants with different occlusions (plants in all groups).

Figure 11 exhibits regression analyses between automatic and manual measurements of scale-related traits of plants with different occlusion (plants in all groups). The average values of leaf area, perimeter, length, and width have values of 2946.10 mm^2^, 216.92 mm, 77.88 mm, and 50.59 mm, respectively. The automatically calculated values are highly correlated with the manually measured values based on the proposed method (EF = 0.9992, EF = 0.9827, EF = 0. 8919, and EF = 0.9039 for the area, perimeter, length, and width, respectively). Additionally, the RMSE values of the area, perimeter, length, and width are 14.12 mm^2^, 4.11 mm, 3.42 mm, and 1.98 mm, respectively. The MAPE values of the area, perimeter, length, and width are 0.72%, 2.69%, 6.83%, and 7.16%, respectively. All the regression analyses and the results of RMSE and MAPE comparing manual measurement traits versus automatic measurement traits for plants with different occlusions show that the accuracy of the proposed morphological trait estimation method is competitive and effective.

#### 3.2.2. Results of Scale-invariant Morphological Traits

Six scale-invariant traits are estimated. The average scale-invariant traits X_11_, X_12_, X_13_, X_14_, X_15_, and X_16_ are 12.93, 36.14, 55.56, 2.79, 4.30, and 1.55, respectively. Table 4 shows the values of EF of regression analyses between the automatic and manual measurement of scale-invariant traits of plants with different occlusions. For plants from the first group to the fourth group, EF for the scale-invariant traits X_11_, X_12_, X_13_, X_14_, X_15_, and X_16_ have values of 0.9883–0.9579, 0.9202–0.8461, 0.9386–0.8276, 0.8728–0.7775, 0.8312–0.6891, and 0.8268–0.6895, respectively.

The measurement accuracy of scale-invariant traits of plants with different occlusions is listed in Table 5. For plants from the first group to the fourth group, the results of RMSE have values of 0.27–0.44, 1.83–3.19, 2.83–4.47, 0.17–0.23, 0.23–0.34, and 0.07–0.14 for traits X_11_, X_12_, X_13_, X_14_, X_15_, and X_16_, respectively. The results of MAPE have values of 1.95–2.65%, 5.30–7.08%, 5.76–6.78%, 4.83–7.07%, 4.56–7.04%, and 3.38–6.19% for traits X_11_, X_12_, X_13_, X_14_, X_15_, and X_16_, respectively. The results of RMSE and MAPE increase with the increase of occlusion degree, which indicates that the measurement accuracy decreases with the increase of occlusion degree. The differences of RMSE and MAPE increase overall from the first group to the fourth group.

For all plants, the automatically calculated values are correlated with the manually measured values based on the proposed method (EF = 0.9811, EF = 0.8908, EF = 0.9162, EF = 0.8509, EF = 0.7838, and EF = 0.7434 for traits X_11_, X_12_, X_13_, X_14_, X_15_, and X_16_, respectively). In addition, the RMSE values of traits X_11_, X_12_, X_13_, X_14_, X_15_, and X_16_ are 0.23, 1.46, 2.09, 0.14, 0.20, and 0.08, respectively, and the MAPE values of the corresponding traits are 2.50%, 6.89%, 6.34%, 6.44%, 6.21%, and 5.52%, respectively.

### 3.3. Time Cost

Table 6 lists the time cost of the experiments. For all the experiments, the data scanning time ranges from 120 to 250 seconds. The output time of a 3D model of a plant is between 5.68 and 6.24 seconds. The data processing time including segmentation and trait estimation varies from 4.02 to 5.13 seconds. Data scanning occupies most of the time. In general, the average measurement time of one plant is 196.37 seconds (186.08 seconds for data scanning, 5.95 seconds for 3D model output, and 4.36 seconds for automatic selection of typical leaf samples and morphological trait estimation).

## 4. Discussion

### 4.1. Measurements on Different Canopy-Occluded Plants

In this paper, a novel method based on a handheld laser scanner to automatically measure the morphological traits of typical leaf samples of different canopy-occluded live plants was proposed. Plants with different canopy occlusions were tested. The experimental results show that the measurement accuracy of the proposed method for automatically estimating morphological traits of leaves is competitive. It should be noted that the measurement accuracy is different when measuring plants with different canopy occlusions.

The presented results of scanning (Table 3) show that the scanning accuracy is decreased with the increase of canopy occlusion of plants. The plants with no and a little canopy occlusion can be well scanned (both with scanning accuracy 100%), and the plants with medium and heavy canopy occlusion can lose some data (with scanning accuracy 96.20% and 89.96%, respectively). This is because plants with heavier canopy occlusion have more attached and overlapped leaves, which makes data scanning more difficult. This problem is common in data acquisition based on non-penetration devices [27,28,33]. 

The presented results of segmentation (Table 3 and Figure 6) show that the segmentation accuracy is decreased with the increase of canopy occlusion of plants. R_2_ varies from 100% to 88.8% R_3_ has values from 100% to 79.95%. There are two reasons. On one hand, the increase of canopy occlusion makes the lack of scanning data increase, which decreases the segmentation accuracy indirectly. On the other hand, the increase of canopy occlusion makes the complexity of plant geometric structure increase, which increases the difficulty of the segmentation algorithm directly. This is the reason that the experiment subjects in most contemporary research ([18,19,23]) are plants with several leaves at the early growth stage when measuring leaf morphological traits. Additionally, the difference between R_2_ and R_3_ shows that the main factor that affects the segmentation results is the incomplete scanning data, which indicates the occlusion is the main factor that affects the segmentation accuracy.

The presented results of morphological trait estimation (Figure 7, Figure 8, Figure 9 and Figure 10 and Table 4 and Table 5) show that the trait estimation accuracy is decreased with the increase of canopy occlusion of the plants. There are two main reasons. On one hand, the occlusion decreases the segmentation accuracy, which influences the trait estimation indirectly. On the other hand, the occlusion leads to less growth space, which influences the geometrical shape of leaves and directly affects the trait calculation algorithms. Regarding the regression analyses between automatic and manual measurements of morphological traits, the values of EF are decreased with the increase of plant canopy occlusion. Specifically, the values of EF for the area, perimeter, length, width, X_11_, X_12_, X_13_, X_14_, X_15_, and X_16_ vary from 0.9997, 0.9861, 0.9132, 0.9156, 0.9883, 0.9202, 0.9386, 0.8728, 0.8312, and 0.8268 to 0.9981, 0.9600, 0.7536, 0.7502, 0.9579, 0.8461, 0.8276, 0.7775, 0.6891, and 0.6895, respectively. The morphological trait measurement of plants with no canopy occlusion has high values of EF, and the plants with heavy canopy occlusion have low values. This illustrates the measurement accuracy of plants with no canopy occlusion is higher than that of plants with heavy occlusion. The values of EF in regression analyses are all positive, which illustrates the automatically calculated values are correlated with the manually measured values. Figure 7, Figure 8, Figure 9 and Figure 10 also illustrate the automatic measurements are underestimated compared to manual measurements. This is because it is easy to lose some data in data scanning and leaf segmentation. Regarding the RMSE and MAPE between the automatic and manual measurements of morphological traits, the values of RMSE and MAPE are increased with the increase of plant canopy occlusion. Specifically, the values of RMSE of the area, perimeter, length, width, X_11_, X_12_, X_13_, X_14_, X_15_, and X_16_ vary from 13.59 mm^2^, 4.73 mm, 4.09 mm, and 2.78 mm, 0.27, 1.83, 2.83, 0.17, 0.23, and 0.07 to 48.13 mm^2^, 8.21 mm, 7.37 mm, 4.41 mm, 0.44, 3.19, 4.47, 0.21, 0.34, and 0.14, respectively. The values of MAPE of the area, perimeter, length, width, X_11_, X_12_, X_13_, X_14_, X_15_, and X_16_ vary from 0.60%, 2.16%, 5.29%, 5.79%, 1.95%, 5.30%, 5.76%, 4.83%, 4.56%, and 3.38% to 0.77%, 2.87%, 7.03%, 6.85%, 2.65%, 7.08%, 6.78%, 7.07%, 7.04%, and 6.19%, respectively. It can be found out that the morphological trait measurement of plants with no canopy occlusion has low values of RMSE and MAPE and the plants with heavy canopy occlusion have high values. The values of RMSE exhibit that the automatic trait estimation accuracy can reach the military level. The values of MAPE of the area and perimeter are smaller than those of length and width in the same canopy occlusion condition. This is because calculation algorithms of length and width are based on the 3D mesh of the leaf. The values of MAPE of the scale-related traits are lower than those of scale-invariant traits. This is because that the scale-invariant traits are derived from the scale-related traits.

It should be noted that varied results between the automatically and manually measured morphological traits are usually found when leaves are attached or overlapped. The deviation becomes larger when there are more connected and overlapped parts. This is because the attached or overlapped leaves make segmentation difficult. What is worse, the leaves with a large area of connected and overlapped parts would fail to be segmented.

The presented time cost (Table 6) shows that the time cost increases with the increase of the canopy occlusion. It can be found out that the main time cost is in the data scanning. Plants with more complicated canopy occlusion require more time for data acquisition. The data processing is really fast and the difference in the time cost of different canopy-occluded plants can be ignored. The low time cost indicates the proposed method is competitive and has the potential for real-time plant measurement.

It can be found out although the measurement accuracies from plants with no canopy occlusion to heavy occlusion are reduced slightly, the overall accuracies of scanning, segmentation, and morphological trait estimation are competitive (Figure 11). The time cost of the proposed method for different canopy-occluded plants is low. The competitive measurement accuracy and low time cost of the proposed method show great potential for real-time plant measurement and high-throughput plant phenotype.

### 4.2. Comparison of Related Methods

**Figure 12:**
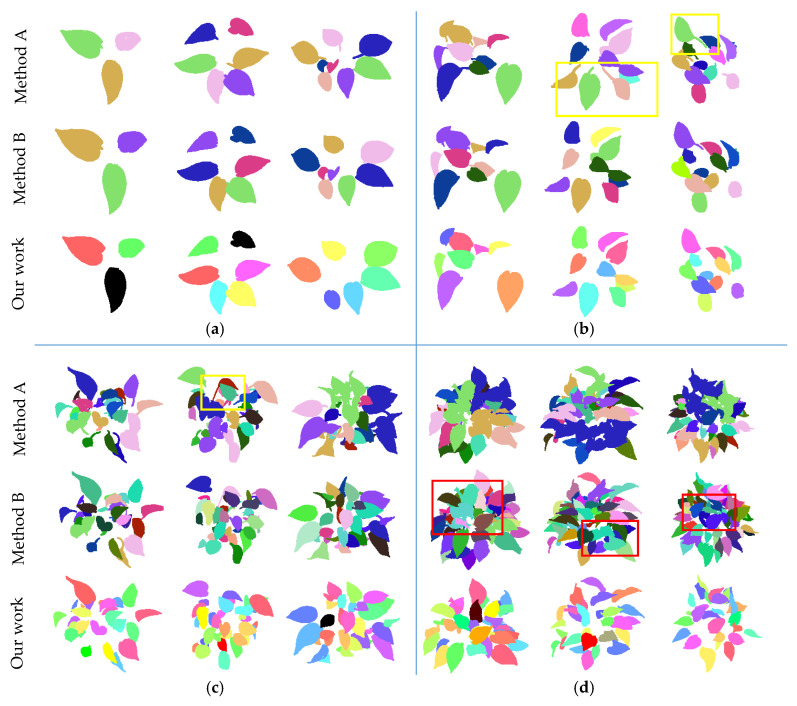
Segmentation results of two segmentation methods and our proposed method. Method A is the Euclidean Clustering method in papers [24,29] and B is the Facet Region Growing method in paper [27]. Plants in (**a**), (**b**), (**c**), and (**d**) are plants with no, a little, medium, and heavy canopy occlusion. The yellow boxes mark some segmented results of leaves with stems using the Euclidean Clustering method. The red boxes mark some segmented results of attached and overlapped leaves using the Facet Region Growing method.

In this paper, a multi-level region growing segmentation using two leaf shape models was proposed to perform individual leaf segmentation and morphological trait estimation. The segmentation comparison of two widely used segmentation methods and our segmentation method is presented in Figure 12. Method A is the Euclidean Clustering method in papers [24,29] and B is the Facet Region Growing method in paper [27]. It can be found out that the Euclidean Clustering method performs well in plants with no and a little canopy occlusion. Some attached leaves are failed to be segmented based on the Euclidean Clustering method when the plants have medium canopy occlusion. Large numbers of leaves are failed to be segmented based on the Euclidean Clustering method when the plants have heavy canopy occlusion. It should be noted that the segmented leaves with the Euclidean Clustering method are usually with stems (the leaves in the yellow boxes in Figure 12), which will affect the leaf measurement accuracy in the morphological trait calculation process. Regarding the Facet Region Growing method, it performs well for plants with no, a little, and medium canopy occlusion. However, some attached and overlapped leaves (the leaves in the red boxes in Figure 12) fail to be segmented using Facet Region Growing method when plants are heavily occluded. It can be seen that the number of leaves that could not be segmented by the Facet Region Growing method is more than ours. It should be noted that the segmentation results using both the Euclidean Clustering method and Facet Region Growing method includes leaves with incomplete scanning data and newborn leaves, which will affect the morphological trait estimation accuracy. It should be noted that the typical leaf samples that are suitable for morphological trait estimation can be automatically selected using our proposed method, while the other two segmentation cannot.

The measurement accuracies of morphological traits using the Euclidean Clustering method, Facet Region Growing method, and our proposed method are listed in Table 7 and Table 8. The values of EF of automatically measured and the manually measured traits using the Euclidean Clustering method, the Facet Region Growing method, and our proposed are increased. The values of RMSE and MAPE using the Euclidean Clustering method, the Facet Region Growing method, and our proposed are decreased. It can be found out that the measurement accuracies using the Facet Region Growing method are higher than those employing the Euclidean Clustering method. The proposed method has higher measurement accuracies compared to the Facet Region Growing method and the Euclidean Clustering method.

### 4.3. Advantages, Limitations, Improvements, and Future Works

In this paper, a novel method for automatic measurement of the morphological traits of typical leaf samples of different canopy-occluded live plants using a handheld laser scanner is proposed. Our method has four advantages. First of all, the 3D mesh model of the plant obtained by ZGScan 717 has higher precision and richer details compared to those reconstructed by the SFM method [24], time-of-flight (ToF) method [22], binocular stereo vision method [27], LIDAR method [34], and FastSCAN hand-held scanner [35]. The 3D mesh model is reconstructed in real-time during data scanning and can be output directly, thus saving data processing time for 3D reconstruction in most contemporary research. Second, typical leaf samples can be automatically selected, and the accuracies of segmentation and trait estimation are competitive compared to related works [27,29,33]. A multi-level region growing segmentation method using two leaf shape models is proposed. The two main segmentation parameters, smooth and curvature, are adaptive to different input data at different segmentation scales. The two shape models, one using scale-related traits and the other using scale-invariant traits, are dynamically established based on the different segmentation results at different scales. The proposed segmentation method performs better compared with the Euclidean Clustering method in papers [24,29] and the Facet Region Growing method in paper [27]. Third, the time cost of our proposed method, especially the data processing time, is really low. Finally, compared with most of the literature [18,19], which only focuses on the research of non-occluded plants at the early growth stage, the method proposed in this paper can be applied to different canopy-occluded plants.

It should be noted that the segmentation method and trait estimation algorithm are based on the 3D mesh models. It has little to do with the acquisition method of the 3D model. Hence, the proposed method in this paper is universal no matter what instrument is used for obtaining 3D mesh models of plants.

However, there are still some limitations in our research. For the process of data scanning, it is necessary to stick the mark points on and around the plant leaves in advance. It should be noted that the proposed method is suitable for plants with a similar leaf shape to Epipremnum aureum, such as eucalyptus, cinnamon, and gardenia. Specifically, the leaves should be broad and elliptical. Leaves with long and banding shapes, such as rice, corn, and wheat, may not be suitable. This is because their leaves are usually soft and curved, which makes the length and width calculation algorithms differ from the elliptical ones.

Thus, the improvement and future work will focus on two problems, namely trait estimation algorithm improvement and segmentation improvement.

## 5. Conclusions

This paper presents a novel method based on a hand-held 3D laser scanner to automatically extract typical leaf samples and estimate their morphological traits from live plants with different canopy occlusions. First, the 3D mesh model of the plant that is reconstructed in real-time during data scanning by a hand-held 3D laser scanner (ZGScan 717) is obtained directly. The plant model has high precision (maximum accuracy of 0.03 mm) and rich details of plant architecture. Then a multi-level region growing segmentation method using two leaf shape models is conducted to automatically extract typical leaf samples and estimate their morphological traits. Particularly, the two main segmentation parameters, smooth and curvature, are adaptive to different plants at different segmentation scales, and the shape model dynamically updates based on the different segmentation results at different scales using the principal component analysis method. Four scale-related traits and six corresponding scale-invariant traits are automatically estimated. Four groups of different canopy-occluded plants are tested. Experiments show that for plants with different canopy occlusions, 94.02% of typical leaf samples can be scanned well and 87.61% of typical leaf samples can be automatically extracted. The automatically measured morphological traits are correlated with the manually measured values (EF = 0.9992, EF = 0.9827, EF = 0. 8919, EF = 0.9039, EF = 0.9811, EF = 0.8908, EF = 0.9162, EF = 0.8509, EF = 0.7838, and EF = 0.7434 for the area, perimeter, length, width, area perimeter ratio, area length ratio, area width ratio, perimeter length ratio, perimeter width ratio, and aspect ratio, respectively). In addition, the RMSE values of those 10 morphological traits are 14.12 mm^2^, 4.11 mm, 3.42 mm, 1.98 mm, 0.23, 1.46, 2.09, 0.14, 0.20, and 0.08, respectively, and the corresponding MAPE values are 0.72%, 2.69%, 6.83%, 7.16%, 2.50%, 6.89%, 6.34%, 6.44%, 6.21%, and 5.52%, respectively. The average time of one plant measurement is 196.37 seconds (186.08 seconds for data scanning, 5.95 seconds for 3D model output, and 4.36 seconds for data processing).

The successful application of the proposed method on four different canopy-occluded plants shows the robustness of the proposed method in plant measurement. Therefore, it can be concluded that our method can automatically select typical leaf samples of live plants with different canopy occlusions and output 10 leaf morphological traits with high measurement accuracy and low time cost. The proposed method demonstrates the ability of rapid batch processing of data, which is potential for real-time plant measurement and high-throughput plant phenotype.

Since occlusion is the main factor affecting measurement accuracy, we are interested in exploring more effective segmentation methods, such as adding color information to improve the segmentation accuracy. We also plan to integrate the proposed method into a hand-held scanner system to achieve real-time plant measurement.

## Figures and Tables

**Table 1:** Parameters of ZGScan 717.

Types	Parameters	Types	Parameters
Weight	1.0 kg	Accuracy	0.03 mm
Volume	310 × 147 × 80 mm	Field depth	550 mm
Scanning area	275 × 250 mm	Transfer method	USB 3.0
Speed	480,000 times/s	Work temperatures	−20–40 °C
Light	7 laser crosses (+1)	Work humidity	10–90%
Light security	Ⅱ	Outputs	Point clouds/3D mesh

**Table 2:** Morphological traits. Sym.: symbols of the traits. Var.: variables.

Scale-Related Traits	Sym.	Var.	Scale-Invariant Traits	Sym.	Var.
Area	*s*	*X*_01_	Area perimeter ratio	*s*/*c*	*X*_11_
Perimeter	*c*	*X*_02_	Area length ratio	*s*/*l*	*X*_12_
Length	*l*	*X*_03_	Area width ratio	*s*/*w*	*X*_13_
Width	*w*	*X*_04_	Perimeter length ratio	*c*/*l*	*X*_14_
			Perimeter width ratio	*c*/*w*	*X*_15_
			Aspect ratio	*l*/*w*	*X*_16_

**Table 3:** Accuracy of data scanning and individual leaf segmentation of typical leaf samples. NO. is the number of plants. *N*_0_ is the number of leaves on the plant. *N*_1_, *N*_2_, and *N*_3_ are the numbers of manually selected, well scanned, and automatically selected typical leaf samples. R_1_, R_2_, and R_3_ are the average value of R__scan_, R__seg1_, and R__seg2_ for each group.

Groups	NO.	*N*_0_	*N*_1_	*N*_2_	*N*_3_	R__scan_	R__seg1_	R__seg2_	R_1_	R_2_	R_3_
1 (No occlusion)	1	3	3	3	3	100.00%	100.00%	100.00%	100.00%	100.00%	100.00%
	2	6	6	6	6	100.00%	100.00%	100.00%			
	3	8	6	6	6	100.00%	100.00%	100.00%			
2 (A little occlusion)	4	10	8	8	8	100.00%	100.00%	100.00%	100.00%	100.00%	100.00%
	5	10	10	10	10	100.00%	100.00%	100.00%			
	6	11	11	11	11	100.00%	100.00%	100.00%			
3 (Medium occlusion)	7	26	24	23	22	95.83%	95.65%	91.67%	96.20%	94.94%	91.33%
	8	32	25	24	23	96.00%	95.83%	92.00%			
	9	34	31	30	28	96.77%	93.33%	90.32%			
4 (Heavy occlusion)	10	45	37	35	32	94.59%	91.43%	86.49%	89.96%	88.80%	79.95%
	11	50	37	33	29	89.19%	87.88%	78.38%			
	12	53	36	31	27	86.11%	87.10%	75.00%			
all	1–12	288	234	220	205	94.02%	93.18%	87.61%	-	-	-

**Table 4:** The values of EF of regression analyses between the automatic and manual measurement of scale-invariant traits for plants with different occlusions.

Groups	X_11_	X_12_	X_13_	X_14_	X_15_	X_16_
1	0.9857	0.9202	0.9102	0.8728	0.8312	0.8268
2	0.9883	0.9130	0.9386	0.8513	0.8147	0.7594
3	0.9692	0.8507	0.9017	0.8472	0.7449	0.7322
4	0.9579	0.8461	0.8276	0.7775	0.6891	0.6895
All	0.9811	0.8908	0.9162	0.8509	0.7838	0.7434

**Table 5:** The measurement accuracy of scale-invariant traits for plants with different occlusions.

Groups	RMSE							MAPE (%)					
	X_11_	X_12_	X_13_	X_14_	X_15_	X_16_		X_11_	X_12_	X_13_	X_14_	X_15_	X_16_
1	0.27	1.83	2.83	0.17	0.27	0.07		1.95	5.30	5.88	4.83	5.66	3.38
2	0.28	2.20	2.95	0.18	0.23	0.10		2.02	6.77	5.76	5.37	4.56	4.54
3	0.38	2.39	3.44	0.23	0.34	0.14		2.65	7.08	6.21	7.07	7.04	6.19
4	0.44	3.19	4.47	0.21	0.31	0.13		2.64	7.06	6.78	6.59	6.21	5.87
All	0.23	1.46	2.09	0.14	0.20	0.08		2.50	6.89	6.34	6.44	6.21	5.52

**Table 6:** Time cost (second). T_scan: time of data scanning. T_mesh: time of 3D model output. T_p: time of data processing.

Plants	Points	T_scan	T_mesh	T_p	Plants	Points	T_scan	T_mesh	T_p
1	203,343	120	5.68	4.02	7	434,177	185	6.08	4.25
2	279,350	130	5.71	4.02	8	400,144	191	6.00	4.27
3	304,232	140	5.81	4.02	9	420,915	199	6.05	4.29
4	364,758	160	5.89	4.08	10	449,642	265	6.12	5.13
5	364,299	170	5.88	4.09	11	456,619	247	6.13	5.04
6	359,396	176	5.81	4.09	12	493,968	250	6.24	5.06

**Table 7:** Measurement accuracies of the scale-related morphological traits using the Euclidean Clustering method, Facet Region Growing method, and our method.

Methods		Area	Perimeter	Length	Width
Method A	EF	0.8430	0.8299	0.7872	0.7501
	RMSE	54.98 mm^2^	18.56 mm	11.44 mm	9.66 mm
	MAPE	5.44%	9.44%	16.03%	18.57%
Method B	EF	0.9354	0.9103	0.8546	0.8212
	RMSE	23.98 mm^2^	12.13 mm	7.12 mm	5.47 mm
	MAPE	2.13%	6.12%	9.12%	11.57%
Our work	EF	0.9992	0.9827	0.8919	0.9039
	RMSE	14.12 mm^2^	4.11 mm	3.42 mm	1.98 mm
	MAPE	0.72%	2.69%	6.83%	7.16%

**Table 8:** Measurement accuracies of the scale-related morphological traits using the Euclidean Clustering method, Facet Region Growing method, and our method.

Methods		X_11_	X_12_	X_13_	X_14_	X_15_	X_16_
Method A	EF	0.8286	0.7812	0.7513	0.7001	0.6108	0.5603
	RMSE	0.78	5.67	7.63	0.45	0.49	0.17
	MAPE	5.17%	14.39%	12.68%	12.99%	12.68%	10.34%
Method B	EF	0.9102	0.8512	0.8417	0.8103	0.7029	0.6819
	RMSE	0.46	3.57	4.09	0.25	0.30	0.10
	MAPE	3.59%	10.47%	9.46%	9.79%	9.56%	7.89%
Our work	EF	0.9811	0.8908	0.9162	0.8509	0.7838	0.7434
	RMSE	0.23	1.46	2.09	0.14	0.20	0.08
	MAPE	2.50%	6.89%	6.34%	6.44%	6.21%	5.52%
